# The effects of gaze-display feedback on medical students’ self-monitoring and learning in radiology

**DOI:** 10.1007/s10459-024-10322-6

**Published:** 2024-03-31

**Authors:** Ellen M. Kok, Diederick C. Niehorster, Anouk van der Gijp, Dirk R. Rutgers, William F. Auffermann, Marieke van der Schaaf, Liesbeth Kester, Tamara van Gog

**Affiliations:** 1 Department of Education, Utrecht University, P.O. Box 80140, 3508 CS Utrecht, The Netherlands; 2 Lund University Humanities Lab, Lund University, Lund, Sweden; 3 Department of Psychology, Lund University, Lund, Sweden; 4 Department of Radiology, University Medical Center Utrecht, Utrecht, The Netherlands; 5 School of Medicine, University of Utah, Utah, USA; 6 Utrecht Center for Research and Development in Health Professions Education, University Medical Center Utrecht, Utrecht, The Netherlands

**Keywords:** Eye tracking, Feedback, Gaze display, Medical image perception, Radiograph interpretation, Self-monitoring

## Abstract

Self-monitoring is essential for effectively regulating learning, but difficult in visual diagnostic tasks such as radiograph interpretation. Eye-tracking technology can visualize viewing behavior in gaze displays, thereby providing information about visual search and decision-making. We hypothesized that individually adaptive gaze-display feedback improves posttest performance and self-monitoring of medical students who learn to detect nodules in radiographs. We investigated the effects of: (1) Search displays, showing which part of the image was searched by the participant; and (2) Decision displays, showing which parts of the image received prolonged attention in 78 medical students. After a pretest and instruction, participants practiced identifying nodules in 16 cases under search-display, decision-display, or no feedback conditions (*n* = 26 per condition). A 10-case posttest, without feedback, was administered to assess learning outcomes. After each case, participants provided self-monitoring and confidence judgments. Afterward, participants reported on self-efficacy, perceived competence, feedback use, and perceived usefulness of the feedback. Bayesian analyses showed no benefits of gaze displays for post-test performance, monitoring accuracy (absolute difference between participants’ estimated and their actual test performance), completeness of viewing behavior, self-efficacy, and perceived competence. Participants receiving search-displays reported greater feedback utilization than participants receiving decision-displays, and also found the feedback more useful when the gaze data displayed was precise and accurate. As the completeness of search was not related to posttest performance, search displays might not have been sufficiently informative to improve self-monitoring. Information from decision displays was rarely used to inform self-monitoring. Further research should address if and when gaze displays can support learning.

Self-monitoring is essential for effective self-regulated learning. Self-monitoring refers to an ‘in-the-moment’ judgment of one’s current skill level in the context of task demands (Eva & Regehr, 2011). It plays a role in the regulation of study behavior (Dunning et al., 2004; Metcalfe & Finn, 2008), and thereby impacts learning outcomes (i.e., performance on subsequent tests). Thus, when self-monitoring is inaccurate, suboptimal study decisions are made and learning outcomes suffer. Monitoring is often not accurate (i.e., the learner’s judgment of their own performance deviates from their actual performance Dunlosky et al., 2016; Eva et al., 2004; Griffin et al., 2019)). For example, one study found that when medical students rated their diagnosis of a radiograph as ‘definitely correct’, their diagnosis was correct in only 69% of cases (Pusic et al., 2015). This is problematic, as there is ample evidence that monitoring accuracy can affect restudy behavior and clinical decisions such as asking for a second opinion (Clayton et al., 2023), and inaccurate monitoring could result in diagnostic errors when they happen in the workplace (Berner & Graber, 2008).

In visual diagnostic tasks such as radiograph interpretation, monitoring is especially difficult, because few overt actions happen that can inform self-monitoring. Furthermore, it was also found that people have trouble remembering where they have looked (Kok et al., 2017; Võ et al., 2016), and radiologists report using a different viewing strategy than what they actually used (Aizenman et al., 2017).

## Gaze displays

A potentially useful intervention to improve self-monitoring accuracy and thereby foster learning of visual diagnostic tasks, such as radiograph interpretation, could be to display participants’ gaze (i.e., what they looked at, as measured with eye tracking, Holmqvist et al., 2011; Kok & Jarodzka, 2017a) recorded during task performance, as feedback after the task. In tasks that require visual search for small low-contrast targets like pulmonary nodules in radiographs, observers must look directly at (i.e., fixate) the object (Kundel et al., 1978). Looking directly at an abnormality is an important (albeit not sufficient) condition for perceiving it (Kok & Jarodzka, 2017a, 2017b).

Errors can result from faulty search, that is, an abnormality is not even looked at, or faulty decision making, that is, an abnormality is looked at, but not recognized as such (Kundel et al., 1978). Gaze data thus provide important information about the effectiveness of visual search in tasks such as radiograph interpretation (Brunyé et al., 2019; Kok & Jarodzka, 2017a, 2017b; Van der Gijp et al., 2017). Furthermore, radiologists have been found to look longer at areas where they miss abnormalities than at areas where they correctly detect an abnormality (Manning et al., 2006). Thus, next to information about the effectiveness of visual search, gaze data also provide important information about visual decision-making. As task performance is difficult to monitor especially in visual tasks and people also have trouble monitoring their search behavior (Kok et al., 2017; Võ et al., 2016), with radiologists often reporting using a different search strategy than they actually used (Aizenman et al., 2017), displaying gaze data as feedback after task performance may facilitate monitoring.

**Fig. 1:**
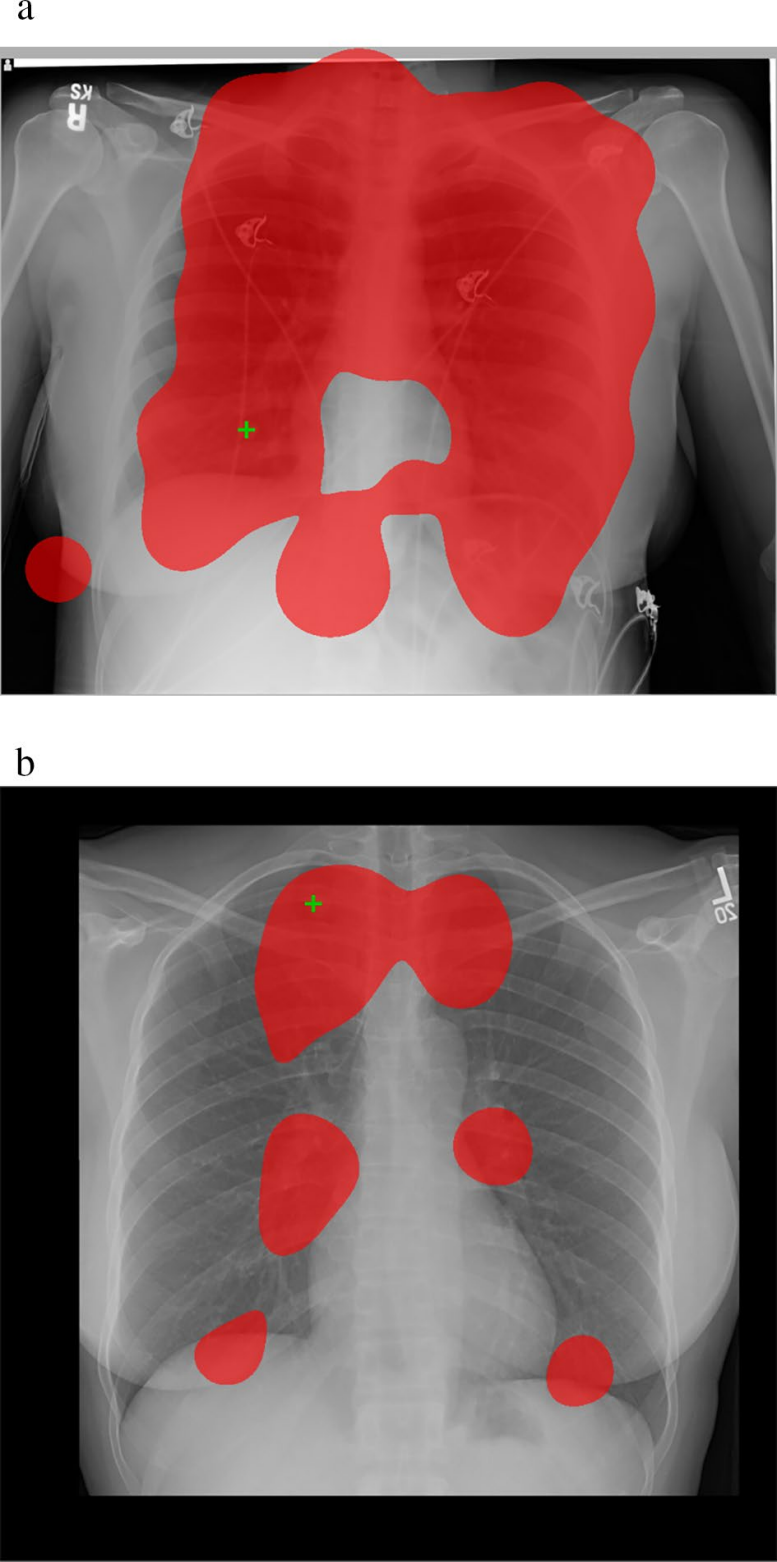
**a***.* Example search display as used in this study that shows which areas (red) are looked at for at least 100 ms (cf. Donovan et al., 2005; Drew & Williams, 2017; Peltier & Becker, 2017). The green cross denotes the location that this participant considered to be abnormal. **b***.* Example decision display as used in this study that shows which areas (red) are looked at for at least 1000 ms (cf. Donovan et al., 2005; Kundel et al., 1990). The green cross denotes the location that the participant considered to be abnormal.

Several studies tested the effectiveness of gaze displays for improving *performance,* i.e., the score on a task in the presence of a gaze display (cf. Soderstrom & Bjork, 2015). In discussing those, we make a distinction between two types of gaze displays: search displays, in which the feedback shows which areas are inspected (even very shortly), and decision displays, in which the feedback shows which areas received prolonged attention (see Fig. 1 for example displays as used in the current study).

Search and decision displays are intended to improve performance in different ways: Search displays are aimed at helping participants avoid search errors. In this case, an abnormality is missed because it is never looked at. By showing which part of the image was searched, gaze displays may support participants not only in checking whether their search was complete but also in helping them to check whether they missed abnormalities because of incomplete search. While intuitively this type of feedback may seem useful, search displays often do not help participants improve their performance (Dickinson & Zelinsky, 2005; Donovan et al., 2005; Drew & Williams, 2017; Eder et al., 2021; Peltier & Becker, 2017). However, several studies found positive effects of decision displays on performance in radiology (Donovan et al., 2008; Krupinski et al., 1993; Kundel et al., 1990). The rationale expressed in those studies is that areas of prolonged fixation (i.e. looked at for a relatively long time) are areas considered as potential abnormalities in the image, but the observer is not necessarily aware of considering those areas. More generally, longer total fixation durations are often taken to reflect that the reader has discrimination difficulties (Holmqvist et al., 2011). While there might be other reasons for prolonged fixations on certain areas (such as mindwandering, e.g., Faber et al., 2020), it has been found that areas that receive prolonged attention are more likely to contain abnormalities than areas that do not receive prolonged attention (Manning et al., 2006; Nodine et al., 2002) even if those abnormalities are not reported. This type of error is not a failure of search but a failure of decision-making and thus they are called decision errors (Kundel et al., 1978). Thus, re-inspecting areas that received prolonged attention might support participants in considering whether a decision error was made.

Together, those studies suggest that gaze displays that visualize search behavior (search displays) are unlikely to improve *performance*. However, displays that show which areas are likely to be difficult in terms of decision-making (decision displays) might positively impact performance. Note that we include a search-display condition in this study anyway to allow for a direct comparison, as such direct comparisons are currently lacking in the literature.

There is only limited insight into the effects of different types of gaze displays on *learning*, i.e., performance on later tasks in the absence of the displays (as measured with a posttest, cf.(Soderstrom & Bjork, 2015) and *monitoring accuracy*; the few available studies were not conducted in medical education. One study (Kostons et al., 2009) found gaze displays to be helpful for participants when they evaluated their performance on genetic problem-solving tasks, whereas another study (Kok et al., 2022) found that gaze displays did not help participants achieve a higher monitoring accuracy in a navigational map-reading task, possibly because the gaze display did not provide participants with information regarding their decision-making. Neither study made a direct comparison of search and decision displays.

To sum up, since gaze displays provide information on visual search and decision-making, learners might be able to use this information to inform their monitoring, and, as a result, adapt their viewing behavior and/or learn to execute the task of nodule detection more effectively.

## The current study

In the current study, we investigate the effects of both search and decision displays as feedback during nodule detection practice on the ability of medical students to identify pulmonary nodules at posttest (i.e., learning), and monitoring accuracy. Participants judged whether they thought that their answer on the presence/absence of nodules was correct or not, and we used this estimation to calculate monitoring accuracy (both globally, for a set of radiographs, and locally, for each case). Monitoring accuracy is defined as the absolute difference between their estimate and their actual test performance (Griffin et al., 2019). Values closer to zero reflect greater monitoring accuracy.

Based on earlier research described above, we expected post-test performance and monitoring accuracy to be higher in the decision-display condition than in the search-display condition and control (no feedback) condition (H1). Participants were also asked to judge their confidence in their answers after each case and we explored the effects on confidence in the correctness of answers by reporting average confidence in correct and incorrect responses (cf. Pusic et al., 2015). Finally, participants reported perceived competence and self-efficacy (people’s beliefs in their capability to execute the task, Bandura, 1977) after the experiment, to allow us to explore if gaze-display feedback affected these overall feelings of ability in the task.

To investigate whether feedback was used during practice and post-test performance, we measured the completeness of search (i.e., to what extent students avoid search errors). We expected that participants in the search-display condition show higher completeness of search compared to the other two conditions (H2). Finally, we asked participants to report how they use the information from the display to inform their self-monitoring, and how participants perceive their usefulness and explored differences between the feedback conditions. As students and practitioners in complex visual tasks consider (completeness of) search to be central in learning those tasks (Eder et al., 2021; Kramer et al., 2019; Subramaniam et al., 2006a, [Bibr CR56][Bibr CR55], 2006b) and thus often teach complete viewing strategies (Auffermann et al., 2015a, 2015b; Auffermann et al., 2015a, 2015b), we expected that participants would perceive search displays to be (especially) useful (H3).

## Methods

### Participants and design

Participants (*N* = 78) were first (*n* = 47), second (*n* = 23), and third (*n* = 8) year medical students (24 male, 54 female), *M*_*age*_ = 20.3, *SD* = 2.9, from a Dutch University. They had limited or no experience with diagnosing pulmonary nodules, but had previously received basic training in chest radiograph interpretation. Furthermore, through their coursework, they should be familiar with pulmonary anatomy, pulmonary pathology, and lung cancer. Pulmonary nodules are a potential image manifestation of lung cancer and this concept should be familiar to most medical students. Participants were recruited from among all medical students during regular classes on radiology and using newsletters. The experiment had a between-subjects design with three conditions, to which participants were randomly assigned: no display (n = 26), search display (n = 26), and decision display (n = 26) conditions. Participants were tested in individual sessions. They participated voluntarily and all provided informed consent. A 12-euro payment was provided after participation. The project was approved by the institutional review board and executed in accordance with the declaration of Helsinki.

### Materials and Measures

#### Apparatus

The experiment was conducted using the SMITE toolbox (Niehorster & Nyström, 2020) in MATLAB (The Mathworks Inc, 2018) version R2018B with Psychtoolbox Version 3.0.16 (Kleiner et al., 2007) and presented on a 22-inch monitor (1680 × 1050 pixels). Eye movements were recorded using an SMI RED250 eye tracker (GmbH, 2017) with a sampling rate of 250 Hz. Questionnaires were presented in Qualtrics (Qualtrics, 2005).

#### Radiographs

32 chest radiographs (16 normal, 16 with a single simulated nodule) were randomly assigned to a pretest (6 cases), practice phase (16 cases), and posttest phase (10 cases) such that the prevalence of nodules was 50% in each phase. A different assignment and stimulus order was made for every set of three participants (one in each condition). This set of radiographs was previously successfully used as training and test material in a similar population (Auffermann et al., 2015a, 2015b). The test performance was computed for each phase as the percentage of correctly diagnosed cases, where a diagnosis was only correct if an abnormality (if present) was clicked on and if healthy tissue was not clicked on (i.e., accuracy).

#### Gaze-display feedback

Gaze displays were generated immediately after a case was looked at in the training phase using a custom script, employing 2D kernel density estimation (Botev et al., 2010) using the locations of fixations (which were classified by the I2MC classification algorithm, Hessels et al., 2017) weighted by their duration and a 2-degree bandwidth. Fixations closer together than 30 pixels and 30 ms were merged. Figure 1 provides examples from the search and the decision condition. Search displays showed all areas looked at for at least 100 ms, in transparent red (60% opacity) overlaid on the original stimulus. Decision-displays showed all areas looked at for at least 1000 ms, again in transparent red overlaid on the original stimulus. Locations that were clicked on by the participant were shown as green crosses. Participants in the control condition would just see the original stimulus and green crosses.

As the effects of gaze displays might depend on the quality of the eye-tracking recording, we developed a dichotomized measure of data quality (high or low) to quantify whether participants saw a high or low-quality gaze display during practice. Data was considered high quality if it adhered to the following three rules: The accuracy in degrees of visual angle was smaller than 1.0˚. Data loss was smaller than 20%. Precision (Root mean square, RMS) was smaller than 2 SD of the whole sample (Holmqvist et al., 2011).

Average accuracy and precision for the full sample and the high-quality sample can be found in Appendix A. Considering those strict guidelines, 16 participants in the search-display condition and 18 participants in the decision-display condition (65%) saw a high-quality gaze display in the practice phase. Note that after each calibration, participants were shown the location of their gaze. Calibration was repeated if they did not consider the location shown to match their actual gaze location, so even low-quality gaze displays provided a relatively good display of viewing behavior.

#### Instruction and training

Participants received written instructions about the detection of pulmonary nodules on chest radiographs consisting of 30 electronic slides with written text and images and including 5 practice images after which a correct answer was provided. Participants could read each slide at their own pace but not move back. The instruction consisted of a general part about nodule detection and how to apply a complete viewing strategy, and a condition-specific part that explained the gaze displays and how they related to viewing behavior (see Appendix B). The general instruction was a translated version of a training that was earlier used for a similar population (Auffermann et al., 2015a, 2015b, 2015a, 2015b). Those prior studies have shown that such perceptual tasks are generally feasible for medical students and other healthcare trainees.

#### Questionnaires

##### Monitoring and confidence judgments

After each phase, participants reported how many cases they thought they had correctly diagnosed during that phase (global monitoring judgment). After each case, participants reported whether they thought that their answer to the case was correct or incorrect (local monitoring judgment) and rated their confidence in their answer (0–100%).

Global absolute accuracy was the difference between the estimated number of cases correctly diagnosed (judgment) and the actual number of cases correctly diagnosed during each phase (performance). To allow for comparison with the local monitoring accuracy, the global absolute accuracy was divided by the number of cases so the range was 0 to 1.

Local monitoring accuracy was calculated per phase as the average of the absolute differences between the estimate (correct or incorrect, 1 or 0) and the correctness of the answer (0 or 1) of each case in that phase (range from 0 to 1 for each phase).

##### Perceived competence

As a post-experimental measure of perceived competence, we used the ‘perceived competence’ subscale of the Intrinsic Motivation Inventory (IMI) (Center for Self-Determination Theory, 2019; Ryan, 1982), which was previously validated (McAuley et al., 1989). This scale has six items and uses a 7-point Likert response format ranging from 1 (not true at all), 4 (somewhat true), to 7 (very true). Internal consistency (as expressed by McDonald’s omega (Hayes & Coutts, 2020)) in our sample was high (*ω* = 0.90). Perceived competence was the average of the six items of the ‘perceived competence’ of the IMI (Ryan, 1982).

##### Self-efficacy

In line with recommendations by Bandura (2006), we measured task-specific self-efficacy by asking “Rate how confident you are that you can detect lung nodules as of now. Rate your degree of confidence by recording a number from 0 to 100 using the scale given below.” We used a 10-point visual analog scale ranging from 0 (cannot do at all), 50 (moderately certain can do), to 100 (highly certain can do).

##### Feedback use and usefulness

After the experiment, participants reported how they used the feedback in response to the question ‘Based on what did you answer the question ‘How sure are you about your diagnosis after evaluation’?’^1^ Two researchers individually coded, with condition blinded, whether participants referred to using the feedback as information about their search process (code: Search), as information about their decision-making process (code: Decision), or whether they referred to using the feedback without explaining further (code: Feedback general). The agreement was acceptable (Krippendorff’s Alpha = 0.73). In case of disagreement about the reported feedback, the code ‘Feedback general’ was assigned.

Participants were also asked to fill out an adapted version of a 5-item questionnaire to measure the perceived usefulness of feedback (Rakoczy et al., 2019), on a 4-point scale from 0 (completely disagree) to 3 (completely agree) with items such as ‘the feedback helped me recognize where I can improve’. Reliability in our sample was high (*ω* = 0.81).The experienced usefulness of the gaze display was the average of the five items of the experienced usefulness questionnaire.

#### Completeness of search measure

For each case in the practice phase and test phase, the percentage of the image fixated for at least 100 ms in each of the cases was calculated, and the average was reported.

### Procedure

Participants first completed a 6-case pretest and provided a local monitoring judgment and confidence judgment after each case. After the six cases, they provided a global monitoring judgment. Next, participants were provided with the instruction slides. Subsequently, the eye tracker was calibrated, and after that, participants practiced a set of 16 cases: They were provided with a radiograph and asked to mark all pulmonary nodules (if any). Participants were informed that they could click on multiple locations, but that no more than one abnormality would be present. They were not informed about the overall prevalence of abnormalities.

**Fig. 2:**
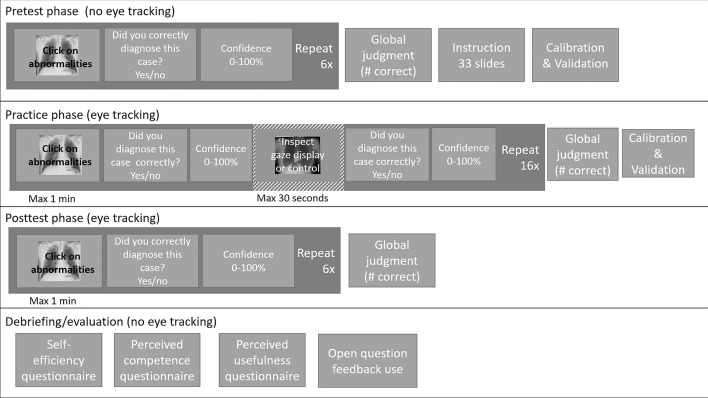
Overview of the different phases of the design. The stripped box (Inspect gaze display or control) indicates the experimental manipulation

Cases were presented for a maximum of 1 min but with the option to proceed earlier. After each case, they were asked to provide a local monitoring judgment and confidence judgment. The intervention was implemented as feedback after practicing each case: All participants saw the case again with a green cross in the location where they had marked abnormalities. Depending on the assigned condition, participants in the gaze-display conditions additionally saw either a search or decision gaze display as feedback. No corrective feedback was provided. After the feedback, they were again asked to provide a local monitoring judgment and confidence judgment. After practicing with all cases, they provided a global monitoring judgment. Next, participants finished a 10-case posttest in which they searched for nodules again, and provided local monitoring and confidence judgments after each case and, after the 10 cases, a global monitoring judgment. Finally, participants were asked to fill in a short questionnaire about their self-efficacy, perceived competence, experienced usefulness of the gaze displays, and their feedback use. See Fig. 2 for an overview of the procedure and Appendix B  for detailed instructions.

### Analyses

#### Gaze data quality

All analyses were executed twice: once with all participants included, and once with only those participants included who saw a high-quality gaze display during practice (as well as all participants in the control condition). We report the results of the analyses for all data in the results section (based on the rationale that this is most authentic; i.e., in practice, you might not always have high-quality data) and the analysis with high data quality in Appendix C, and report it in the results section when the high-quality data only gives a different pattern of results.

#### Statistical analyses

Data were analyzed using Bayesian ANOVAs (van den Bergh et al., 2020) and t-tests using JASP version 0.17.3 (JASP Team, 2023). We used default settings unless specified differently. Inclusion Bayes factors (*BF*_*inclusion*_) were reported to qualify the evidence in the data for including the condition as a predictor of the outcome variable. For example, *BF*_*inclusion*_ = 3 means the data are three times more likely under the alternative hypothesis (i.e. the condition is a substantial predictor) than under the null hypothesis (i.e. no differences between conditions) whereas *BF*_*inclusion*_ = 0.3 means that the data are three times more likely under the null-hypothesis than under the alternative hypothesis (Marsman & Wagenmakers, 2017).

## Results

### Pre-analyses

Table 1 provides the descriptive statistics for the dependent variables. Bayesian ANOVAs on pretest data showed reasonable evidence that there was no difference between conditions in pretest performance and global absolute accuracy at pre-test. For local absolute accuracy, however, there is evidence for a difference between conditions, with monitoring being most accurate in the decision-display condition and least accurate in the search-display condition.

**Table 1:** Average Test Performance, Global Absolute Accuracy, Local Absolute Accuracy, Self-efficacy, Perceived Competence, Completeness, and Perceived Usefulness by Phase and by Condition

Condition	*Range*	Control		Search-display		Decision-display		*BF*_*inclusion*_
		*M*	*SD*	*M*	*SD*	*M*	*SD*	
*Test Performance*								
Pretest	0–100%	28.8%	20.3%	23.7%	19.0%	26.9%	19.5%	0.16
Practice	0–100%	63.5%	11.3%	61.1%	19.1%	52.6%	16.8%	1.33
Posttest	0–100%	69.2%	17.0%	65.0%	15.3%	60.0%	16.5%	0.56^1^
*Global monitoring accuracy*								
Pretest	0–1	0.21	0.17	0.24	0.17	0.23	0.14	0.15
Practice	0–1	0.25	0.14	0.25	0.19	0.17	0.17	0.39
Posttest	0–1	0.27	0.17	0.22	0.20	0.20	0.19	0.25
*Local monitoring accuracy*								
Pretest	0–1	0.46	0.19	0.54	0.30	0.33	0.24	4.76^2^
Practice: estimate before feedback	0–1	0.36	0.14	0.41	0.16	0.37	0.16	0.21
Practice: estimate after feedback	0–1	0.34	0.11	0.41	0.17	0.36	0.15	0.35
Posttest	0–1	0.31	0.13	0.35	0.17	0.35	0.17	0.17^3^
*Self-efficacy*	0–100	41.15	14.51	43.08	20.74	45.80	16.04	0.16
*Perceived competence*	1–7	3.25	0.90	3.22	1.12	3.43	0.85	0.15
*Completeness*								
Practice	0–100%	43.34%	7.34%	49.78%	7.19%	45.69%	7.76%	1.12
Posttest	0–100%	40.56%	8.33%	46.91%	8.75%	42.50%	8.89%	0.70
*Perceived usefulness*	0.2–3			1.91	0.58	1.55	0.63	1.68^4^

Test performance was the percentage correct on the pretest (6 cases), during practice (16 cases), and on the posttest (10 cases). For monitoring accuracy, lower scores denote more accurate monitoring. For Completeness, only data of participants with high-quality data is included. *M* = Mean, *SD* = Standard deviation, *BF*_*inclusion*_ = Inclusion Bayes factor. Superscripts denote lines where the inclusion Bayes Factor when only high-quality data is included in the analysis differs substantially from the reported inclusion Bayes Factor. ^1^*BF*_*inclusion*_ = 0.23*, *^*2*^*BF*_*inclusion*_ = 1.19*,* ^3^*BF*_*inclusion*_ = 0.359*,* ^4^*BF*_*inclusion*_ = 18.63

### Effects of gaze displays on posttest performance and monitoring accuracy

In contrast to hypothesis 1, the Bayesian ANOVA showed uncertainty as to whether there is a difference between conditions in the post-test performance or not. However, note that even if there is a difference between conditions, the score is highest in the control condition. When only the post-test performance of participants who saw high-quality gaze displays was included, somewhat stronger evidence against a difference between conditions was found.

Furthermore, and also in contrast to hypothesis 1, we found evidence against an effect of condition on both the global and local monitoring accuracy at posttest. When only the post-test performance of participants who saw high-quality gaze displays was included, the evidence against an effect of condition on local monitoring accuracy was somewhat weaker.

As local monitoring judgments were made before and after feedback, we additionally used a repeated-measures Bayesian ANOVA with the factor condition and the factor time to take a somewhat more fine-grained look at the effects of condition on local monitoring. This also allowed for including local monitoring accuracy at the pretest in the analysis, and for looking at differences between the practice phase and the posttest. Thus, the factor time had four levels (pretest, during practice before feedback, during practice after feedback, posttest).

When running this analysis on all data, evidence for an interaction of time with condition is uncertain (*BF*_*inclusion*_ = 1.61), and also the main effect of condition is uncertain (*BF*_*inclusion*_ = 0.98). There is evidence for a main effect of time (*BF*_*inclusion*_ = 9.39). Post hoc comparisons show evidence for a difference in monitoring accuracy between the pre and post-test (*BF*_*inclusion*_ = 5.75). On average, monitoring accuracy was somewhat better in the post-test than in the pretest, but numerically, this was not true for the decision-display condition. There was also evidence against a difference in monitoring accuracy between the estimate before and after the feedback in the practice phase (*BF*_*inclusion*_ = 0.25), and between the estimate after feedback and during the posttest (*BF*_*inclusion*_ = 0.26). For all other comparisons, the evidence is uncertain in either direction. Note that individual comparisons are based on default t-tests without corrections for multiple testing as this is not available in JASP.

When running this analysis on the dataset with only participants who saw a high-quality gaze display, there was evidence against an interaction of time with condition (*BF*_*inclusion*_ = 0.23), uncertainty regarding the main effect of condition (*BF*_*inclusion*_ = 1.13) and uncertainty regarding the main effect of time (*BF*_*inclusion*_ = 1.54).

Overall, and in contrast to hypothesis 1, it seems that monitoring accuracy is not positively affected by the presence of gaze displays. To further explore monitoring accuracy and the potential influence of feedback, we report the average confidence in correct and incorrect responses before and after feedback in Table 2. Appendix D provides a detailed overview of how often participants changed their estimated correctness. The pattern looks very similar between conditions: Even though confidence is different in correct versus incorrect responses, it is generally low, and average confidence hardly changes based on the feedback. Overall, participants were unlikely to change their estimated correctness and changed only in 9.7% of cases. This would not be problematic if estimates made before the feedback would already be accurate. However, this was true for only 58% of the cases.

**Table 2:** Average confidence (%) in Correct and incorrect Responses Before and After Feedback by Condition

	Control		Search Display		Decision Display	
	Before (%)	After (%)	Before (%)	After (%)	Before (%)	After (%)
Confidence in correct responses	55.4	55.3	51.5	51.7	51.8	51.4
Confidence in incorrect responses	46.0	43.9	41.3	41.3	43.2	42.0

### Effects of gaze displays on perceived competence and self-efficacy

There was evidence against the effect of conditions for both self-efficacy and perceived confidence (see Table 1). In all conditions, both self-efficacy and perceived competence were rather low.

### Feedback use and perceived usefulness

**Fig. 3:**
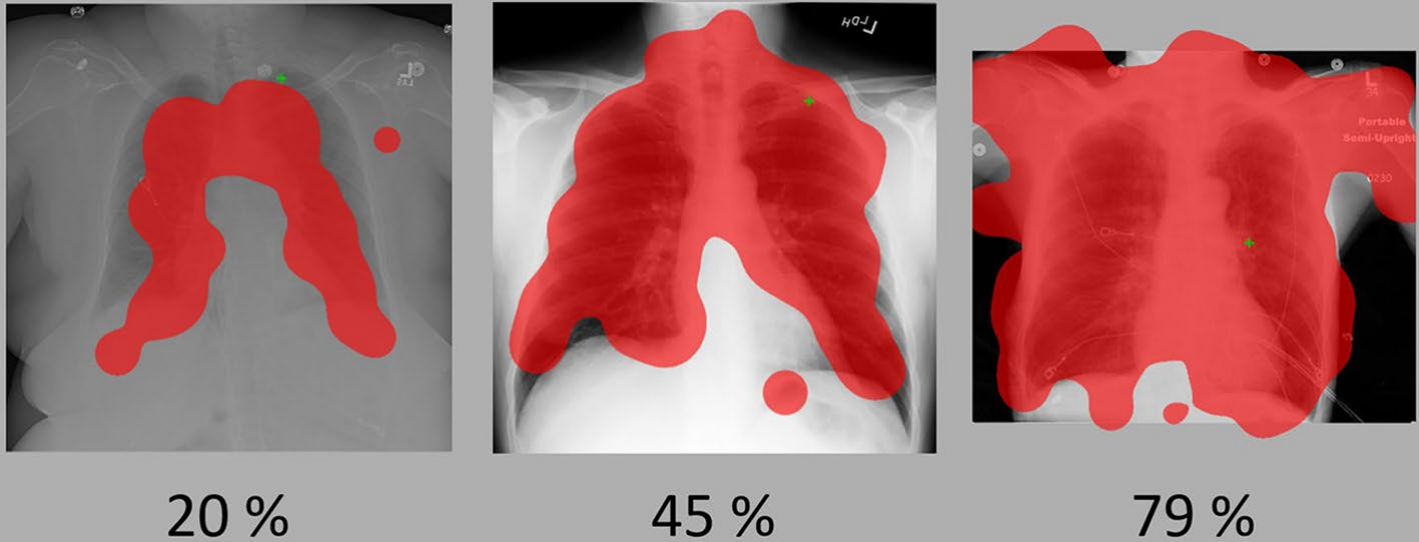
Gaze displays of participants in the search-display condition with lowest (20%), average (45%), and highest (79%) completeness. The first two gaze displays are classified as low data quality, the third gaze display shows high data quality

We expected that participants in the search-display condition show higher completeness of search compared to the other two conditions (H2). The evidence regarding the effect of condition on completeness of search, however, was uncertain for the practice phase, and there was evidence against an effect of condition for completeness of search in the test phase. The correlation between completeness and the score was minimal for both the practice phase (*r* = − 0.09, *BF*_*inclusion*_ = 0.19) and the test phase (*r* = − 0.04, *BF*_*inclusion*_ = 0.19). This reflects that the average completeness of search and test performance were not related, and thus that completeness of search is not predictive of the score. To explore this finding, we inspected all gaze displays of participants in the search-display condition, and present those with the lowest, average, and highest completeness in Fig. 3. It was found that practically all lung tissue was fixated for at least 100 ms in all cases. Indeed, only one abnormality was never fixated in the practice phase (decision condition), and two abnormalities were never fixated in the test phase (search condition).

Table 3 presents the analysis of the answers to the question about feedback use. Whereas many participants in the search-display condition indeed used the gaze display to provide them with information about their search process, only a few participants in the decision-display condition seem to have used the gaze display to inform them about their decision processes. Note that some participants in the decision-display condition interpreted the display as providing them with search information.

**Table 3:** Number and percentage of participants in each condition who reported using the feedback

	Control		Search Display		Decision Display	
	n	%	n	%	n	%
Search information	0	0	17	65.4	4	15.4
Decision information	0	0	0	0	4	15.4
Feedback general	1	3.8	7	26.9	12	46.2
Other	25	96.2	2	7.7	6	23.1

*N* = 26 per condition

### Effects of gaze displays on perceived usefulness and feedback use

There was evidence against an effect of condition for perceived usefulness. However, when only data of participants with high-quality data in the practice phase was considered, participants in the search-display condition rated the usefulness higher (*M* = 2.13, *SD* = 0.48, *n* = 16) than participants in the decision-display condition (*M* = 1.47, *SD* = 0.63, *n* = 18), *BF*_*inclusion*_ = 18.6, which was in line with hypothesis 3.

## Discussion

In this experiment, we used eye-tracking technology to generate gaze-display feedback that showed participants’ search behavior or areas of potential faulty decision-making. In contrast to hypothesis 1, gaze displays did not have positive effects on post-test performance, and global and local monitoring accuracy. They also did not affect perceived competence and self-efficacy. In contrast to hypothesis 2, the completeness of search was not higher in the search-display condition, search was effectively complete in all conditions. Participants in the search-display condition reported using the gaze display feedback more often to inform their self-monitoring than the participants in the decision-display condition (but this did not lead to higher monitoring accuracy). In line with hypothesis 3, search displays were perceived to be more useful than decision displays, but only if they showed high-quality data. Overall, the training supported a substantial improvement in nodule detection performance from pre to post-test, but there was no difference between the conditions.

Interestingly, confidence in correct responses was close to 50% in all conditions, reflecting that participants were rather unsure of their performance, and when participants were correct, the gaze display did not reduce doubt in their answers. When participants were incorrect, their confidence was somewhat lower but still substantial (around 40%) and the gaze display did not lead to lower confidence (see Table 2 and Appendix D for more details).

Thus, participants may not have used the gaze displays in a way that could have helped them improve their performance and self-monitoring, or the information provided by the gaze displays was not related to performance. The gaze displays were designed to provide information about either search or decision-making, and participants were informed about the information provided and how this relates to their viewing behavior (See Appendix B). However, only a few participants reported using the decision display to infer information about their decision-making process.

Participants reported using the information from the search display more often, but participants generally already executed a complete viewing strategy in their initial search, as can be seen in Fig. 3. Thus, like in earlier research, the instruction on how the apply a complete viewing strategy was effective in establishing a complete search (Auffermann et al., 2015a, 2015b; Auffermann et al., 2015a, 2015b). However, in this study completeness was not correlated with the test performance in the practice phase (cf. Kok et al., 2016; Van Geel et al., 2017). That means that even though participants used the feedback to infer information about their search strategy, this search strategy was not predictive of their performance and therefore, did not help to improve monitoring accuracy. At the same time, information about decision-making was often not used by participants. In earlier studies, it was found that this information was predictive of test performance (see Donovan et al., 2008; Krupinski et al., 1993; Kundel et al., 1990. Similar calculations could not be executed in this study).

A similar pattern was found in a navigational map-reading task (Kok et al., 2022). Participants also mostly interpreted the gaze displays (no distinction was made in search/decision display) in terms of the effectiveness of their search instead of using the information to infer difficulties in decision-making. Likewise, completeness of search *alone* was not predictive of learning. This also explains the difference with work by Kostons and colleagues (2009). In this study, participants executed problem-solving tasks in biology (heredity calculations based on Mendel’s law) and saw their gaze afterward. In their task, the gaze displays provided feedback on how effectively the problem-solving steps were executed, and (although they do not report it) it is likely that this was in fact predictive of task performance. However, Kostons et al. (2009) did not look at the effects of gaze displays on monitoring accuracy so a direct comparison cannot be made. Overall, it appears that the effect of gaze-display feedback is contingent on whether the display conveys information that is predictive of task performance, and on whether participants interpret the display as such. An interesting avenue for further research is the development of gaze displays in collaboration with learners to make sure they convey information that helps them interpret their task performance.

## Limitations

Several factors impact the generalizability of the findings in this study. First of all, the focus of this study was on a nodule detection task, which differs from other visual diagnostic tasks in its reliance on search (Kok et al., 2012), and the effects of gaze displays might not generalize to other tasks. Likewise, we tested a specific design of the gaze display. It has been argued that design choices might impact the effectiveness of a gaze display (Emhardt et al., 2023; Kok et al., 2023) so other types of displays might still be effective for fostering self-monitoring. Furthermore, our focus in this study was on novice learners and our findings might thus not generalize to more experienced participants. Participants in our sample mostly interpreted those gaze displays as providing them with information on the completeness of their search process. As their search was mostly complete, gaze displays did not foster monitoring and learning. However, it is yet unclear whether participants with more prior knowledge might be better able to interpret the displays and thus benefit from them: More experienced radiologists might interpret gaze displays more in terms of the decision process (cf. Kundel et al., 1990; Krupinski et al., 1993). Conversely, the performance of more experienced participants might also be harmed by the presence of gaze displays. Experts rely less on a complete viewing strategy for visual search, as, according to Kundel’s model of holistic image perception (Kundel et al., 2007), they form a quick holistic impression that guides subsequent search. A search display fosters completeness of search, and might be especially distracting for them (cf. the expertise reversal effect, Kalyuga, 2009). Further research could investigate the effectiveness of gaze displays to support monitoring and learning in more experienced participants. Finally, participants did not have previous experience with the task of gaze-display interpretation. Although we provided participants with instructions regarding the meaning of the display, Table 3 shows that participants did not always interpret the decision displays as intended. Further research could investigate the effects of gaze displays in different tasks (e.g., volumetric images), different gaze-display designs, or a longitudinal study in which participants gain experience with gaze-display interpretation.

## Conclusion

Neither search nor decision gaze-display feedback had a positive effect on posttest performance, global and local monitoring accuracy (during practice and post-test), perceived competence, and self-efficacy. Participants in the search-display condition reported using the search-display feedback more often than participants in the decision-display condition reported using the decision-display feedback. However, the completeness of the search was not related to test performance in both the practice and posttest phase, and it could therefore be argued that search displays did not provide diagnostic information for self-monitoring. Decision-making was (likely) related to performance, so interventions to foster decision-making should be further investigated (Kok et al., 2016; Kramer et al., 2019). Since self-monitoring was generally inaccurate and confidence was not well-calibrated (i.e., participants did not have very high confidence in correct answers nor very low confidence in incorrect answers), our findings do show that there is a need for interventions to foster self-monitoring among medical students when learning radiology.

## Appendix A: Descriptive statistics of data quality measures in the practice phase

**Table:**

	All Data				High-Quality data			
	Search display		Decision display		Search display		Decision display	
	*M*	*SD*	*M*	*SD*	*M*	*SD*	*M*	*SD*
Accuracy	1.31	1.94	0.89	0.42	0.72	0.14	0.67	0.17
Precision	0.16	0.26	0.10	0.04	0.10	0.04	0.10	0.05
*N*	26		26		16		18	

## Appendix B: Participant instructions

Apart from general information about nodule detection, the instructions provided to participants included a short discussion of Kundel’s model of holistic image perception and discusses that in radiograph interpretation, a large part of the image is inspected, but only for a short time (< 0.1 s). Some areas are inspected in more detail (up to a second or more) because they are suspicious and might contain abnormalities. Participants were informed that areas that are considered ambiguous generally receive longer attention. Subsequently, the instruction differed per condition.

Participants in the decision display condition were informed that they receive gaze displays as feedback, which show in red all areas that have been looked at relatively long (at least 1 s), and they were informed that “A high percentage of missed nodules received prolonged attention although the reader may not be consciously aware that he/she considered them. This feedback is designed to help decrease this sort of errors.” (cf. Kundel et al., 1990).

Participants in the search-display condition were informed that they receive gaze displays as feedback, which show in red all areas that they have looked at, even very shortly (at least 0.1 s) will be colored red, and they were informed that “A high percentage of missed nodules received prolonged attention although the reader may not be consciously aware that he/she considered them. This feedback is designed to help decrease this sort of errors.” (cf. Drew & Williams, 2017).

Participants in the control condition were informed that they will see the same image again, and “It has been found that errors are made when people instantly continue their task without pauses for evaluating their performance. This evaluation moment is designed to help decrease this sort of errors.”

## Appendix C: Results with only high-quality data included

See Table

**Table 4:** Average Test Performance, Global Absolute Accuracy, Local Absolute Accuracy, Self-efficacy, Perceived Competence, Completeness, and Perceived Usefulness by Phase and by Condition

Condition	Control		Search-display		Decision-display		
	*M*	*SD*	*M*	*SD*	*M*	*SD*	*BF*_*inclusion*_
*Test Performance*							
Pretest	28.8%	20.3%	28.1%	19.0%	27.8%	20.6%	0.139
Practice	63.5%	11.3%	60.2%	19.1%	52.1%	16.0%	1.302
Posttest	69.2%	17.0%	66.3%	14.6%	63.3%	16.8%	0.233
*Global monitoring accuracy*							
Pretest	0.21	0.17	0.23	0.18	0.25	0.17	0.183
Practice	0.25	0.14	0.26	0.19	0.15	0.16	0.960
Posttest	0.27	0.16	0.23	0.20	0.23	0.19	0.192
*Local monitoring accuracy*							
Pretest	0.46	0.19	0.52	0.34	0.32	0.24	1.192
Practice before feedback	0.36	0.14	0.41	0.15	0.36	0.16	0.247
Practice after feedback	0.34	0.11	0.43	0.15	0.35	0.15	0.680
Posttest	0.31	0.13	0.39	0.19	0.31	0.18	0.359
*Self-efficacy*	41.15	14.51	44.38	19.65	45.00	16.18	0.179
*Perceived competence*	3.25	0.90	3.33	0.96	3.36	0.85	0.147
*Completeness*							
Practice	43.34%	7.34%	49.78%	7.19%	45.69%	7.76%	1.118
Posttest	40.56%	8.33%	46.91%	8.75%	42.50%	8.89%	0.700
*Perceived usefulness*			2.13	0.48	1.47	0.63	18.632

Test performance was the percentage correct on the pretest (6 cases), during practice (16 cases) and on the posttest (10 cases). Self-efficacy was measured on a scale of 0—100, perceived competence was measured on a scale of 1—7, and perceived usefulness was measured on a scale of 0.2—3. *M* = Mean, *SD* = Standard deviation, *BF* = Inclusion Bayes factor. *N*_*control condition*_ = 26, *N*_*Search-display condition*_ = 16, *N*_*Decision-display condition*_ = 18

^4^.

## Effects of gaze displays on self-monitoring measures

To take a somewhat more fine-grained look at effects of condition on local monitoring accuracy, we used a repeated-measures Bayesian ANOVA with the between-subjects factor condition and the within-subjects factor time (pretest, before feedback, after feedback, posttest). When running this analysis on the dataset with only participants who saw a high-quality gaze display, there was evidence against an interaction of time with condition (*BF*_*inclusion*_ = 0.23), uncertainty regarding the main effect of condition (*BF*_*inclusion*_ is 1.13) and uncertainty regarding the main effect of time (*BF*_*inclusion*_ = 1.54).

See Table

**Table 5:** Average confidence (%) in Correct and incorrect Responses Before and After Feedback by Condition

	Control		Search Display		Decision Display	
	Before (%)	After (%)	Before (%)	After (%)	Before (%)	After (%)
Confidence in correct responses	55.4	55.3	53.9	53.3	51.6	51.0
Confidence in incorrect responses	46.0	43.9	41.0	41.2	42.7	41.4

^5^.

## Effects of gaze displays on completeness

As we used only high-quality gaze data for calculating completeness, this section is identical to the one reported in the manuscript.

## Appendix D-I Confidence before and after feedback and change in confidence by condition, split out by correctness of judgment

**Table:**

	Control			Search Display			Decision Display		
	Before	After	Change	Before	After	Change	Before	After	Change
*Judgment not changed*									
Underestimate	32.39	34.11	1.72	25.02	24.11	− 0.91	26.62	26.27	− 0.35
Correct estimate	56.04	56.34	0.3	52.94	53.57	0.63	49.3	48.84	− 0.46
Overestimate	56.67	57.24	0.57	55.25	56.25	1	60.11	60.54	0.43
*Judgment was changed*									
Correct to overestimate	40.09	42.73	2.64	31.5	42.67	11.17	37	52.71	15.71
Correct to underestimate	54.69	29.94	− 24.75	38.71	32.43	− 6.28	49.75	29.75	− 20
Overestimate to Correct	49	33.55	− 15.45	45.11	24.11	− 21	43.5	27	− 16.5
Underestimate to Correct	41	51.13	10.13	27.2	35.2	8	29.5	51.5	22
Total	52.25	51.47	− 0.78	48.2	48.33	0.13	47.92	47.11	− 0.81

An overestimate would be when a participant indicated thinking that the case was correctly diagnosed, but this was not true. An underestimate would be when the participant indicated thinking that the case was not correctly diagnosed when in fact it was.

## Appendix D-2 Frequency of judgment changes

**Table:**

	Control		Search		Decision	
	Frequency	Percentage (%)	Frequency	Percentage (%)	Frequency	Percentage (%)
*Judgment not changed*						
Underestimate	44	11	63	15	55	13
Correct estimate	239	57	233	56	249	60
Overestimate	70	17	93	22	81	19
*Judgment was changed*						
Correct to overestimate	11	3	6	1	7	2
Correct to underestimate	16	4	7	2	8	2
Overestimate to Correct	20	5	9	2	14	3
Underestimate to Correct	16	4	5	1	2	0
Total	416	100	416	100	416	100

## Footnotes

1 Participants also answered this question before the feedback, this data is not considered in the current paper.
